# Origami and 4D printing of elastomer-derived ceramic structures

**DOI:** 10.1126/sciadv.aat0641

**Published:** 2018-08-17

**Authors:** Guo Liu, Yan Zhao, Ge Wu, Jian Lu

**Affiliations:** 1 Department of Mechanical and Biomedical Engineering, City University of Hong Kong, Kowloon, Hong Kong, PR China.; 2 Centre for Advanced Structural Materials, City University of Hong Kong, Shenzhen Research Institute, 8 Yuexing 1st Road, Shenzhen Hi-Tech Industrial Park, Nanshan District, Shenzhen, PR China.

## Abstract

Four-dimensional (4D) printing involves conventional 3D printing followed by a shape-morphing step. It enables more complex shapes to be created than is possible with conventional 3D printing. However, 3D-printed ceramic precursors are usually difficult to be deformed, hindering the development of 4D printing for ceramics. To overcome this limitation, we developed elastomeric poly(dimethylsiloxane) matrix nanocomposites (NCs) that can be printed, deformed, and then transformed into silicon oxycarbide matrix NCs, making the growth of complex ceramic origami and 4D-printed ceramic structures possible. In addition, the printed ceramic precursors are soft and can be stretched beyond three times their initial length. Hierarchical elastomer-derived ceramics (EDCs) could be achieved with programmable architectures spanning three orders of magnitude, from 200 μm to 10 cm. A compressive strength of 547 MPa is achieved on the microlattice at 1.6 g cm^−3^. This work starts a new chapter of printing high-resolution complex and mechanically robust ceramics, and this origami and 4D printing of ceramics is cost-efficient in terms of time due to geometrical flexibility of precursors. With the versatile shape-morphing capability of elastomers, this work on origami and 4D printing of EDCs could lead to structural applications of autonomous morphing structures, aerospace propulsion components, space exploration, electronic devices, and high-temperature microelectromechanical systems.

## Teaser

The breakthroughs on origami and 4D printing of ceramics could turn a new page for structural application of ceramics.

## INTRODUCTION

Shape-morphing assembly, typically driven by capillary force (*1*), mechanical inductor (*2*), shape memory mechanism (*3*), or frontal photopolymerization (*4*), is desirable for a diversity of applications such as robotics (*5*), life science (*6*), biomaterials (*7*), and four-dimensional (4D) printing (*8*, *9*). To date, various materials, including polymers (*2*, *3*, *8*), metals (*2*, *10*), ceramics (*10*, *11*), as well as graphene (*12*) and silicon (*2*), have emerged in shape-morphing assembly. However, ceramic structures derived from soft precursors that allow elastic deformation remained undiscovered.

Polymer-derived ceramics (PDCs), prepared through thermolysis of polymeric ceramic precursors, exhibit remarkable properties of conventional ceramics such as high thermal stability, chemical resistance to oxidation and corrosion, and mechanical resistance to tribology. The microstructures and properties of PDCs can be tuned through tailored polymer systems and thermolysis conditions (*13*). Additive manufacturing of ceramic precursors or polymerized composites is a state-of-the-art technology to construct complicated ceramic (*14*, *15*) or glass (*16*) architectures. Currently, the printing of soft matter is driving innovation in manufacturing (*17*). However, the existing ceramic precursors are not flexible and stretchable. Thus, we develop silicone rubber matrix nanocomposites (NCs) that can be printed and deformed into complex-shaped elastomer structures, as well as transformed into mechanically robust elastomer-derived ceramics (EDCs).

## Results and discussion

**Fig. 1.**
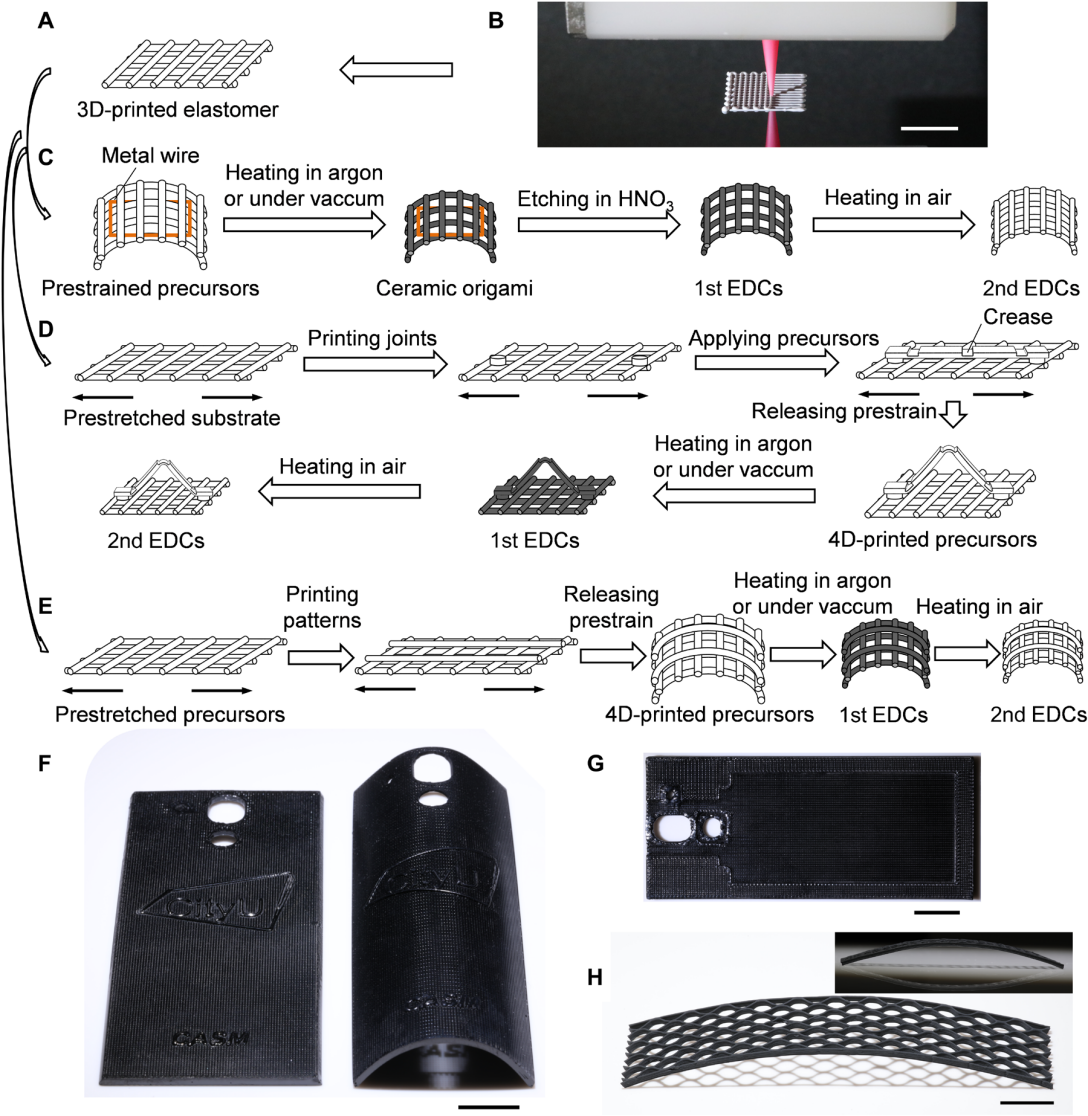
Origami and 4D printing of EDCs via DIW–morphing–heat treatment method. (**A**) 3D-printed elastomeric lattices for origami. (**B**) Optical image of DIW of inks. (**C**) Origami of ceramic structures derived from 3D-printed elastomers. (**D** and **E**) Two 4D printing methods, including method 1 (D) and method 2 (E), together with heat treatment, convert 3D-printed elastomer into 4D-printed ceramics. Examples: (**F**) Flat (left) and curved (right) cellphone back plate. (**G**) Top view of 3D-printed flat cellphone back plate. (**H**) Curved ceramic honeycomb. The inset indicates the curvature of the honeycomb. Scale bars, 1 cm.

The novel silicone rubber NCs were ZrO_2_ nanoparticle (NP)–reinforced poly(dimethylsiloxane) (PDMS). PDMS, the dominant elastomer in silicone systems (*18*), demonstrates its potential as ceramic precursors (*19*), as well as its flexibility (*20*) for origami folding. Furthermore, because of the stretchability (*21*) of PDMS, self-shaping assembly becomes promising. Crystalline ZrO_2_ NPs with a primary average size of 20 to 50 nm in diameter (fig. S1) were incorporated into the PDMS matrix, forming a jammed network within the polymer matrix (*22*), which improves the structural stability and eliminates the shrinkage upon ceramization (fig. S2) (*23*). We developed two ink systems with different types of PDMS, and the amount of ZrO_2_ NPs was 40 weight % (wt %) and 20 wt % in ink system 1 and ink system 2, respectively. 4D printing of EDCs was achieved by the DIW–morphing–heat treatment method (Fig. 1) and started with a cost-effective 3D printing method called direct ink writing (DIW) (Fig. 1B). The 3D-printed elastomer (Fig. 1A), together with the metal wire or stretch device, was prepared for shape morphing. The shape-morphing step could be versatile, including origami and 4D printing. Take the simplest bending configuration as an example. The ceramic precursors were deformed as a function of the prestrain. In origami (Fig. 1C), the metal wire was etched in HNO_3_ after the elastomer-to-ceramic transformation, and chemical resistance of ceramics to corrosion guaranteed the structural stability during the etching process. In 4D printing via method 1 (Fig. 1D), the patterned joints and creases, together with programmed prestrains of the substrate, determined resultant morphology of 3D-printed ceramic precursors on the top. In 4D printing via method 2 (Fig. 1E), designed patterns were printed on the prestretched precursors. Then, programmable self-shaping was implemented with the release of elastic energy stored in precursors. Furthermore, to reveal color versatility in resultant ceramics, we applied a two-step method to develop EDCs. This method converted PDMS NCs into first EDCs via heating in argon or under vacuum, followed by heating in air, yielding second EDCs. The second step was to obtain ceramics with different colors, comparing to those ceramics that underwent the single first step. First EDCs, as precursors of second EDCs, were indispensable to form dense ceramics (fig. S3). Various complex-shaped structures were made with the DIW–morphing–heat treatment method, including cellphone back plate (Fig. 1, F and G) and honeycomb (Fig. 1H).

**Fig. 2:**
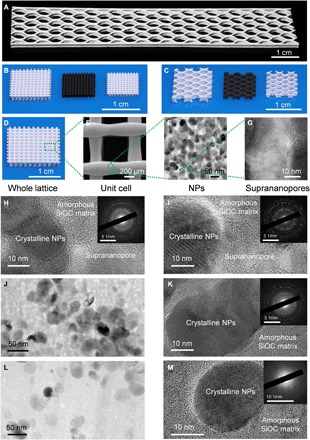
Additive manufacturing of EDCs. (**A**) 3D-printed large-scale elastomeric honeycomb. (**B** and **C**) 3D-printed microlattices (B) and honeycombs (C) of PDMS NCs and first EDCs and second EDCs (left to right). (**D** to **G**) Hierarchical structures of second EDCs illustrated by a digital photo (D), SEM image of the microlattice (E), TEM image of NPs (F), and suprananopores (G). (**H** and **I**) TEM images and SAED patterns (inset) of first EDCs (H) and second EDCs (I) illustrating amorphous-crystalline dual-phase SiOC matrix NCs with suprananopores. The above first EDCs were obtained by heat treatment of ink system 1 at 1000°C under argon flow, and second EDCs were obtained by heat treatment of first EDCs at 1000°C in air. (**J** and **L**) TEM image of first EDCs obtained by heat treatment of ink system 1 at 1300°C (J) and ink system 2 at 1300°C (L), illustrating rare suprananopores. (**K** and **M**) TEM image and SAED patterns (inset) of first EDCs obtained by heat treatment of ink system 1 at 1300°C (K) and ink system 2 at 1300°C (M), illustrating amorphous-crystalline dual-phase SiOC matrix NCs.

Representative periodic structures, including lattices and honeycombs, were fabricated using the DIW–heat treatment method (Fig. 2). The scalability of the DIW method was demonstrated by a large-area elastomeric honeycomb (Fig. 2A). Isotropic shrinkage in the transformations caused good shape retention (Fig. 2, B and C). For ink system 1, heat treatment of first EDCs in air at 1000°C was accompanied by 2.4% mass loss and 2.5% linear shrinkage, while heat treatment of PDMS NCs in argon or under vacuum at 1000°C was accompanied by 35% mass loss and 20% linear shrinkage. Scanning electron microscopy (SEM) observation showed that the spatial resolution of printed ceramic microstructures (Fig. 2D) achieved 200 μm (Fig. 2E), which was determined by the nozzle diameter and the shrinkage during transformation. In the cross section of resultant ceramic lattices, NPs with a diameter of 20 to 50 nm were observed (Fig. 2F). The degradation of the PDMS matrix with well-distributed NPs in ink system 1 yielded porous ceramics with Brunauer-Emmett-Teller (BET) surface areas of 184 and 138 m^2^ g^−1^ for first and second EDCs, respectively (fig. S4). Transmission electron microscopy (TEM) images and pore size distribution of EDCs indicated that suprananopores with a diameter of 2 to 10 nm (Fig. 2G and fig. S4) were found. The suprananoporous ceramics, integrated with printed microstructures, could provide high flux and desired pore accessibility, for example, in ceramic catalyst support. The programmable structural hierarchy of the abovementioned EDCs spanned three orders of magnitude, from 200 μm to 10 cm. The resultant ceramics were amorphous-crystalline dual-phase silicon oxycarbide (SiOC) matrix NCs, as ascertained by TEM images and selected-area electron diffraction (SAED) patterns (Fig. 2, H and I). The resultant ceramics had an average composition of SiO_2.58_C_1.28_Zr_0.29_ and SiO_7.43_C_1.31_Zr_1.91_ for first and second EDCs, respectively, while the average composition of the amorphous matrix in first and second EDCs was SiO_2.14_C_1.44_ and SiO_2.38_C_0.69_, respectively, as measured by energy-dispersive spectroscopy (EDS). First EDCs obtained by heat treatment of ink system 1 and ink system 2 in argon at 1300°C illustrated rare suprananopores (Fig. 2, J and L). The resultant ceramics were also amorphous-crystalline dual-phase SiOC matrix NCs (Fig. 2, K and M) and had an average composition of SiO_1.59_C_0.81_Zr_0.20_ and SiO_0.35_C_0.03_Zr_0.13_, respectively.

**Fig. 3:**
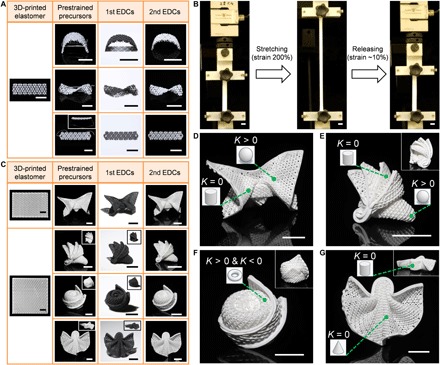
Complex origami of EDCs with mixed Gaussian curvature (*K*). (**A**) Flexibility and stretchability of printed elastomer presented by bending, twisting, and stretching (top to bottom). (**B**) Optical images of printed elastomer from 0 to 200% strain and then to ~10% strain. (**C**) Representative metal wire–assisted ceramic origami mimicking a butterfly, the Sydney Opera House, a rose, and a dress (top to bottom). The insets indicate the location of constrains. (**D** and **E**) Positive *K* (spherical caps) and zero *K* (cylinders) in ceramic origami. (**F**) Positive *K* (the outer region of the torus) and negative *K* (the inner region of the torus) in ceramic origami. (**G**) Zero *K* (cones and cylinders) in ceramic origami. The above first EDCs were obtained by heat treatment of ink system 1 at 1000°C in argon (A) or under vacuum (C), and second EDCs were obtained by heat treatment of first EDCs at 1000°C in air. Scale bars, 1 cm.

The flexibility and stretchability of the printed elastomer, as demonstrated by some fundamental loading modes, such as bending, twisting, and stretching, suggested possibilities for origami assembly (Fig. 3A). The printed ceramic precursors can be stretched beyond three times their initial length (Fig. 3B and movies S1 and S2). Representative ceramic origami structures with complex curvatures, mimicking a butterfly, the Sydney Opera House, a rose, and a dress, were built from 3D-printed precursors (Fig. 3C). The 3D-printed elastomeric lattices underwent morphological transformation and were deterministically constrained by the metal wire, yielding ceramic origami. The printed periodic local patterns in elastomeric lattices improved the foldability of global structures and guaranteed the programmability in locating constraints. The equilibrium morphologies were determined by balancing bending and stretching energies (*24*), and this competition resulted in a thickness-dependent equilibrium state (*25*). On the basis of Gauss’s theorema egregium (*26*), the stretchability of the elastomer offers opportunities to building sophisticated structures with mixed Gaussian curvature (*27*) by changing distances between points on the surface (*24*). Typical zero Gaussian curvature, such as cylinders and cones, and positive Gaussian curvature, such as spherical caps, were generated in ceramic origami, and negative Gaussian curvature was exhibited in the saddle-shaped inner region of the torus (Fig. 3, D to G). The DIW–morphing–heat treatment method allowed versatile transformations, including elastomers/ceramics, soft/hard, large/small, black/white, and 3D printing/4D morphing. Hybrid 3D printing and robotics also hold potentials for improving this processing and product functionality (*28*), for example, automatically applying programmable metal wires in the 3D-printed elastomer for origami. Likewise, kirigami assembly (*29*) can be integrated in the DIW–heat treatment method as another powerful and customizable approach for 4D-printed ceramics. Hence, the DIW–heat treatment method shows a promising future as a module to lock elastomeric behaviors and time in ceramics.

**Fig. 4:**
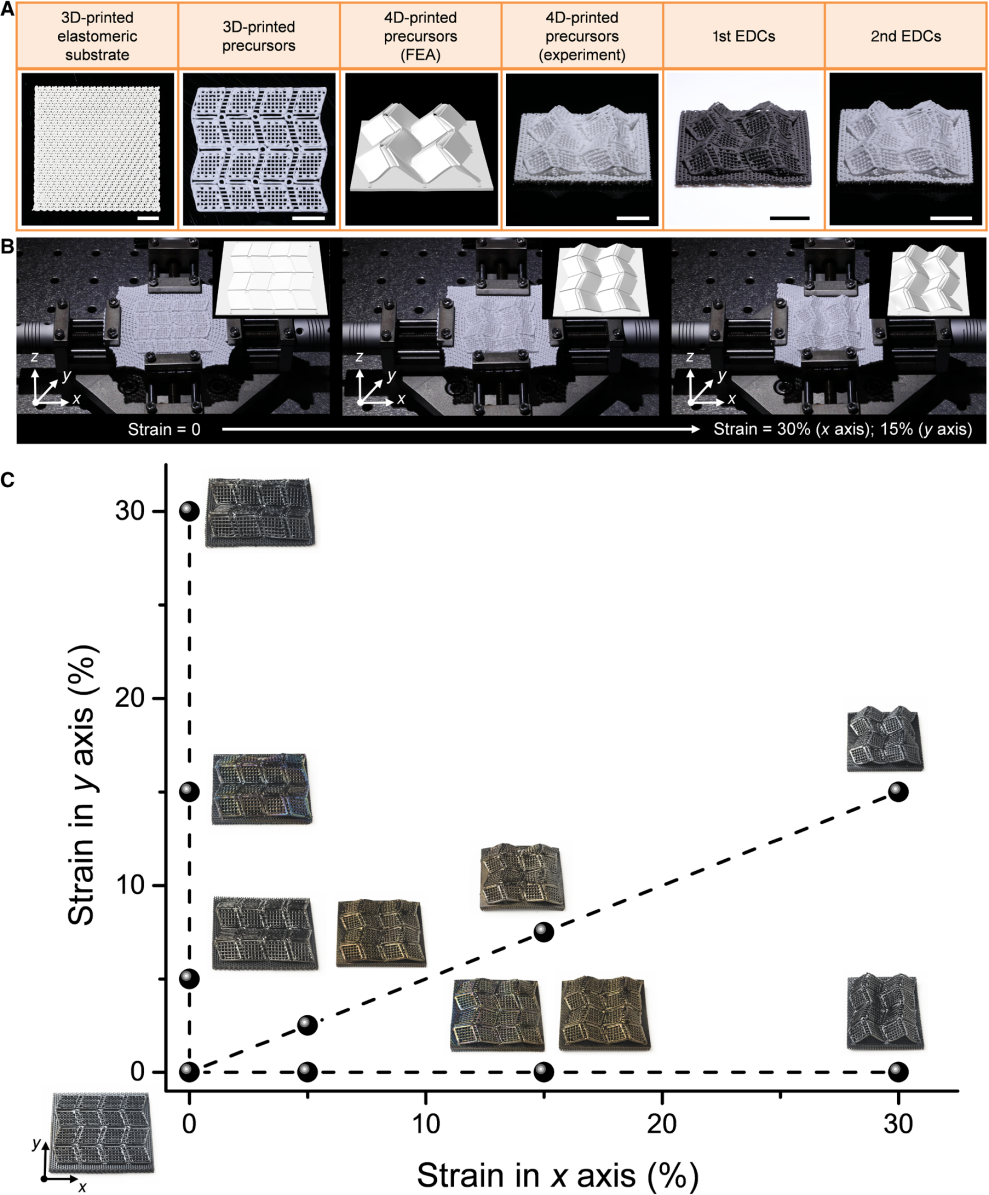
4D printing of EDCs via method 1. (**A**) Representative 4D-printed EDCs with the Miura-ori design. (**B**) 4D printing process of ceramic Miura-ori with a maximum compressive strain of 30% in the *x* axis and 15% in the *y* axis. The above first EDCs were obtained by heat treatment of ink system 1 at 1000°C under vacuum, and second EDCs were obtained by heat treatment of first EDCs at 1000°C in air. (**C**) Phase diagram (^1^/_4_) of 4D printing of Miura-ori as an example to illustrate that a series of complex-shaped ceramics with continuously variable geometries can be derived from a simple design. The series of ceramic Miura-ori were obtained by heat treatment of ink system 2 at 1300°C in argon. Scale bars, 1 cm.

**Fig. 5:**
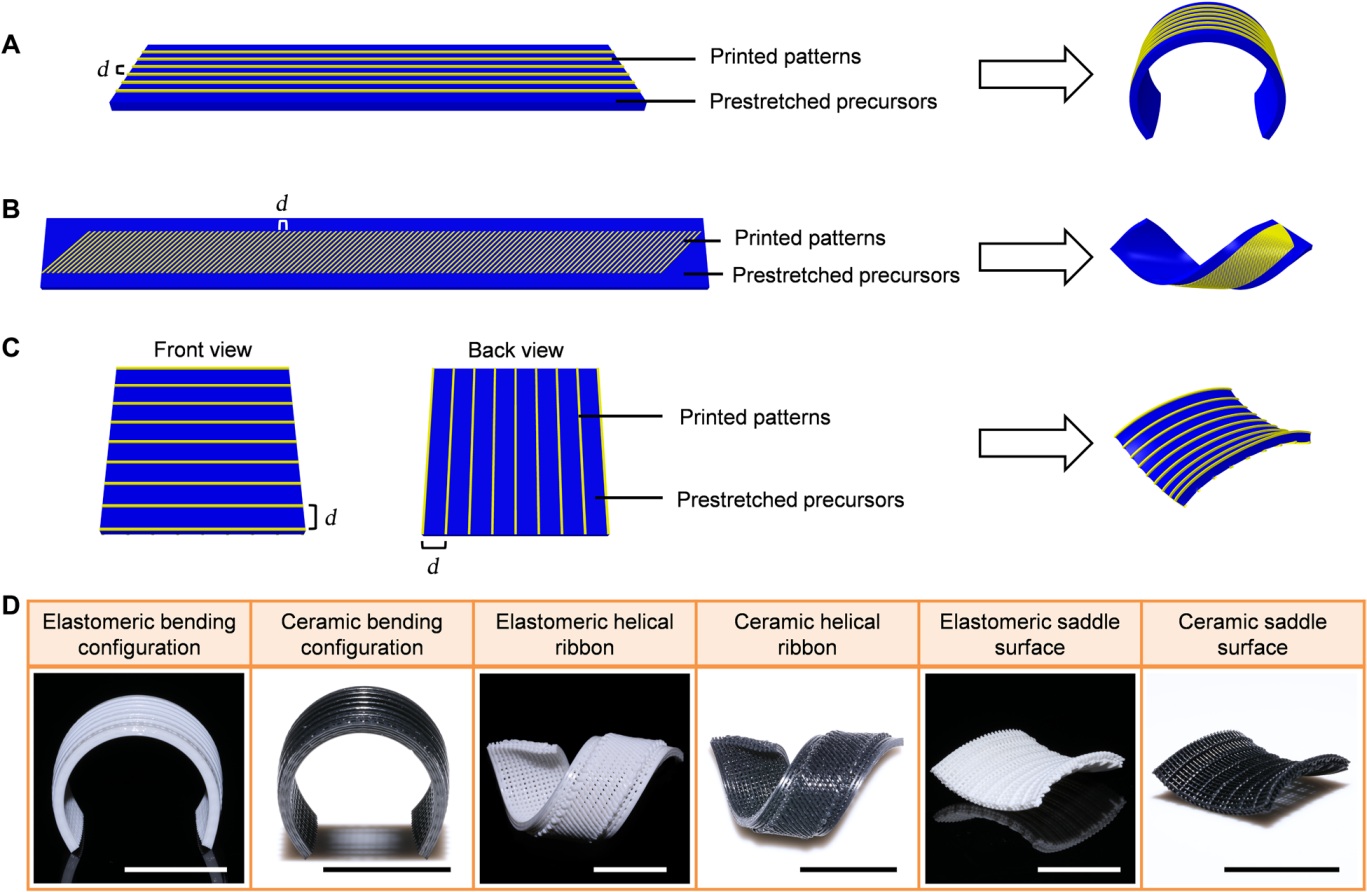
4D printing of EDCs via method 2. (**A** to **C**) 4D printing of ceramics with the design of bending configuration (A), helical ribbon (B), and saddle surface (C), and the definition of important geometric parameters [(A) *d* = 1.2 mm; (B) *d* = 0.7 mm, α = 45°; (C) *d* = 2.5 mm]. (**D**) Representative 4D-printed EDCs by printing programmed patterns on prestretched precursors. Ceramic structures were obtained after heat treatment at 1300°C in argon. Scale bars, 1 cm.

Then, we developed two methods with the self-transformation process to illustrate the possibility of 4D printing EDC structures. In method 1, the Miura-ori design (fig. S5), as a kind of classic topological patterns (*30*), was grown with the assistance of a homemade biaxial stretch device. The substrate and Miura-ori patterns were 3D-printed for this compressive buckling-induced self-morphing process. The important geometric parameters of Miura-ori were illustrated in fig. S5. The ceramic precursors had well-designed creases on the surface, which had lower bending stiffness that enabled easy folding deformation of the structure. The substrate was prestretched by a homemade stretch device, and the stretch device was controlled by four stepping motors, whose rotation rates could be programmably controlled. Then, the cuboid joints connecting the substrate and the Miura-ori patterns were printed, and the printed Miura-ori pattern was applied on the substrate with patterned joints. Afterward, releasing prestrains in the substrate caused compressive buckling of the Miura-ori patterns. The experimental results and finite element analysis (FEA) predictions showed good agreement (Fig. 4, A and B, and movie S3). Because of its periodicity and symmetry, Miura-ori can also serve as the elementary geometric construction for engineering more complex-shaped origami structures (*27*). In method 2, inspired by the opening of plant seed pods (*11*, *31*), representative topological structures, including bending configuration, helical ribbon, and saddle surface, were achieved by printing designed patterns on the printed precursors with previously stored elastic energy (Fig. 5 and movies S4 to S6). For bending configuration, the patterns printed on the prestretched precursors were aligned in the same direction of the stretch force. For helical ribbon, the patterns printed on the prestretched precursors were aligned at well-defined angles of 45° relative to the direction of stretch force. For saddle shape, the patterns were printed on two sides of prestretched precursors. The important geometric parameters were illustrated in Fig. 5. The self-morphing process was programmed by controlling the releasing process of previously stored elastic energy in printed elastomers, as shown in movies S4 to S6.

When it comes to some situations, for example, a series of complex-shaped ceramics with similar geometries were required, this 4D printing concept will show its cost efficiency in terms of time, because a series of complex-shaped ceramics with continuously variable geometries can be derived from a simple design (Fig. 4C and fig. S6). Various 4D printing methods can provide great freedom in designing geometrically complex ceramics that are almost impossible to create by any other method. Furthermore, shape-morphing capabilities of stretchable elastomers can improve the adaptability of structural materials to versatile application environments (*32*), for example, space exploration. 3D-printed elastomeric precursors can be folded to save space and then spread into desired structures. After elastomer-to-ceramic transformation, 4D-printed ceramics can be used as thermal-resistant structures.

**Fig. 6:**
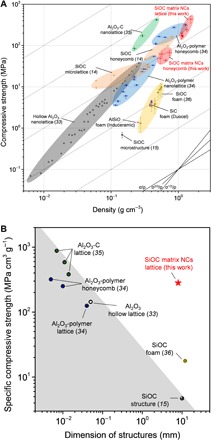
Mechanical robustness of printed EDCs. (**A**) Compressive strength-density Ashby chart. SiOC matrix NC architectures with high specific compressive strength in this work (red stars) were compared with other ceramic structures, as reported in the reference, and commercially available SiC foam and AlSiO foam. For our SiOC matrix NC architectures, the tests were repeated at least three times. Error bars denoted the SDs of the compressive strength values and densities. Please refer to tables S1 and S2 for detailed information. (**B**) Strength-scalability synergy is achieved. Architectured EDCs with simultaneous high strength and large scale in this work (red stars) are separated from other reported ceramic structures. The scalability of each additive manufacturing technique is exhibited by the maximum dimension of resultant ceramic structures, as reported in the reference. The data representing each reference work were selected from all the reported compression data and exhibited the maximum specific compressive strength for a series of samples with the same maximum dimension. For our SiOC matrix NC architectures, the data point represents the average value obtained from 15 samples. Please refer to table S2 for detailed information.

To characterize the mechanical robustness of these ceramic architectures, compression tests were performed on printed ceramic lattices and honeycombs, for both first and second EDCs, and the results were summarized in Fig. 6 and tables S1 and S2. Ceramic structures in this work overcame the strength-scalability trade-off in printed ceramics and were distinguished from previous works, including 3D-printed SiOC microstructures (*15*) and ceramic/ceramic composite nanostructures constructed by 3D laser lithography and atomic layer deposition (*33*–*35*). Unlike most of the reported works using 3D laser lithography (*33*–*35*), our large-scale ceramic architectures are of interest for mechanical systems beyond the laboratory for it overcame the challenge of scalability. A compressive strength of 547 MPa was achieved on the lattice structure at 1.6 g cm^−3^, and the specific compressive strength of our built EDCs was ~19 times as high as conventional accessible SiOC foam (*36*). These light yet strong hierarchical ceramic structures have great potential for the fabrication of multiscale mechanical metamaterials (*37*). Another advantage was that the DIW–heat treatment method was cost-effective when compared with other additive manufacturing techniques for ceramics because the DIW–heat treatment method did not require expensive laser or ultraviolet energy for 3D lithography techniques or sintering of ceramic powders, which required high temperatures above 1600°C (for SiC and Si_3_N_4_) (*15*).

## CONCLUSIONS

The presented method can extend and be further developed to apply to other binary and multiple component systems. These prestrained EDC structures, based on an additive manufacturing technique, will bring substantial benefits to a wide range of fields including aerospace propulsion (*38*) and bio-inspired tough ceramic/organic hybrid materials (*39*, *40*). With the breakthroughs achieved in this development of complex-shaped and mechanically robust ceramic origami and 4D-printed ceramics, more innovations and possibilities in ceramic-related applications could be generated.

## MATERIALS AND METHODS

### Preparation of inks

For ink system 1, liquid PDMS (XE15-645, Momentive Performance Materials) was formulated by mixing a PDMS prepolymer and a curing agent at a 1:1 weight ratio. The ink mixture was manually blended by a glass rod for 30 min. ZrO_2_ NPs [40 wt % (11 volume %); Tong Li Tech Co. Ltd.] were then added. After manually blending or mixing by the triple roller mills (EXAKT 80E) for 2 hours, the ink mixture was poured into a printing syringe and degassed for 2 hours at room temperature. The ink was printable for over 8 hours at room temperature, and its printability could last for over half a year if stored in a refrigerator at −80°C (Thermo Scientific).

For ink system 2, liquid PDMS (SE 1700 Clear, Dow Corning) was formulated by mixing a PDMS prepolymer and a curing agent at a 10:1 weight ratio. The ink mixture was manually blended by a glass rod for 30 min. ZrO_2_ NPs (20 wt %; Tong Li Tech Co. Ltd.) were then added, mixed by the triple roller mills (EXAKT 80E) for 2 hours, and poured into a printing syringe. Afterward, the ink was centrifuged to remove gas bubbles.

### Additive manufacturing of PDMS NCs

The 3D printing of PDMS NC architectures was conducted using a 3D printer (Regenovo Biotechnology Co. Ltd.). For ink system 1, the printing plate was equipped with a heating plate with a temperature of 140° to 150°C. For ink system 2, the ink was printed at room temperature. Each ink housed in a syringe was extruded through a nozzle (diameter, 260 or 410 μm) with a controlled gas pressure and then deposited onto the printing plate. The gas pressure was in the range of 0.2 to 0.6 MPa at a printing speed of 10 to 50 mm s^−1^ and adjusted according to ink behaviors and the nozzle diameter. The height of each printed layer was typically set as 0.8 times of the nozzle diameter to achieve good adhesion between printed layers.

For ink system 1, the deposited ink was rapidly solidified and could be easily removed from the heating plate. A final post-cure was performed at 150°C for 15 min (ink system 1) or 30 min (ink system 2) to guarantee sufficient degree of cross-linking, which was necessary to limit undesired shape changes during heat treatment. The 3D-printed polymer composites in Fig. 2 (B and C) were cut into approximately 1 cm × 1 cm in area before heat treatment.

### Synthesis of EDCs by heat treatment

For ink system 1, 3D-printed PDMS NC structures were heated to 1000°C in a tube furnace with an argon flow of 20 ml min^−1^ or under vacuum followed by natural cooling to ambient temperature in the furnace, and the heating rate was 10°C min^−1^. Heat treatment at 1000°C in an argon flow of 20 ml min^−1^ was accompanied by a working vacuum pump. The obtained first EDCs from heat treatment at 1000°C were then heated to 1000°C at a heating rate of 10°C min^−1^, followed by natural cooling to ambient temperature in a furnace in air, yielding second EDCs. First EDCs were also obtained by heating PDMS NC lattices to 1300°C for 1 hour in a tube furnace with an argon flow of 20 ml min^−1^, and the heating and cooling rates were both 5°C min^−1^.

For ink system 2, first EDCs were obtained by heating PDMS NCs to 1300°C for 5 hours in a tube furnace with an argon flow of 20 ml min^−1^, and the heating and cooling rates were both 1°C min^−1^. Second EDCs were obtained by heating first EDCs to 1300°C for 5 hours in a tube furnace in air, and the heating and cooling rates were both 5°C min^−1^.

### Origami of EDCs

The origami structures in Fig. 3 (A and C) were printed by ink system 1. Six-layer triangular patterns (3 cm × 1 cm × 0.2 cm; nozzle diameter, 410 μm; center-to-center ligament spacing, 2.2 mm) were printed for the bending, twisting, and stretching and then manually folded with the assistance of iron wires (wire diameter, 0.5 mm). The length of the iron wire used in each example was about 6 cm. Six-layer triangular patterns (6 cm × 6 cm × 0.2 cm; nozzle diameter, 410 μm; center-to-center ligament spacing, 1 mm) were printed for the origami structure mimicking the Sydney Opera House, a rose, and a dress in Fig. 3C, and the size of the similar triangular pattern for the origami mimicking a butterfly was 6 cm × 4 cm × 0.2 cm. The complex-shaped origami structures (Fig. 3C) were achieved by manual folding with the assistance of copper wires (wire diameter, 0.2 mm). The length of the iron wire used in each example was about 4 cm. The metal wires were manually threaded through the folded 3D-printed lattice, locating constrains to support the elastomeric origami. The samples used in the metal wire–assisted origami were heated to 1000°C in a tube furnace in argon (Fig. 3A) or under vacuum (Fig. 3C) followed by natural cooling to room temperature in the furnace, and the heating rate was 10°C min^−1^. After heat treatment, first EDCs and metal wires were put in a beaker filled with 30 wt % HNO_3_. The metal wires were removed after about 10 min. First EDCs were cleaned by deionized water and then underwent heat treatment in air to obtain second EDCs.

### 4D printing of EDCs via method 1

The Miura-ori structures in Fig. 4 (A and B) were printed by ink system 1. A nine-layer triangular pattern (6 cm × 6 cm × 0.3 cm; nozzle diameter, 410 μm; center-to-center ligament spacing, 1 mm) was printed as the substrate for the 3D-printed Miura-ori patterns. The overall height of the three-layer parallelogram pattern was ~0.7 mm, and the one-layer crease was printed between the patterns. Furthermore, the Miura-ori design was filled with square patterns (nozzle diameter, 410 μm; center-to-center ligament spacing, 1 mm). The cuboid joints (3.5 mm × 0.9 mm × 0.3 mm) connecting the substrate and the Miura-ori patterns were printed using the same prepolymer inks. Then, the whole device was heated at 150°C for 15 min to solidify the joints. During the releasing process, the rotation rates of the stepping motors in *x* and *y* directions were 2.43 and 1 rotation per second, respectively; hence, the releasing speeds of the substrate in *x* and *y* directions were 4.86 and 2 mm s^−1^, respectively. The above first EDCs were obtained by heat treatment of ink system 1 at 1000°C under vacuum, and second EDCs were obtained by heat treatment of first EDCs at 1000°C in air. The Miura-ori structures in Fig. 4C were printed by ink system 2 with a similar process.

### 4D printing of EDCs via method 2

The structures in Fig. 5D were printed by ink system 2. For bending configuration, the precursor filled with a square pattern (60 mm × 10 mm × 1 mm; center-to-center ligament spacing, 0.5 mm) was printed and then stretched to 30% strain. The patterns printed on the prestretched precursors were filled in a cuboid of 50 mm × 9 mm × 0.21 mm, and the center-to-center ligament spacing was 1.2 mm. For helical ribbon, the precursor filled with a square pattern (60 mm × 10 mm × 1 mm; center-to-center ligament spacing, 0.5 mm) was printed and then stretched to 120% strain. The patterns printed on the prestretched precursors were filled in a cuboid of 90 mm × 6 mm × 0.21 mm, and the center-to-center ligament spacing was 0.7 mm. For saddle shape, the precursor filled with a square pattern (60 mm × 60 mm × 1 mm; center-to-center ligament spacing, 0.5 mm) was printed and then stretched to 20% strain. The patterns printed on two sides of prestretched precursors were both filled in a cuboid of 44 mm × 44 mm × 0.21 mm, and the center-to-center ligament spacing was 2.5 mm. The nozzle diameter was 260 μm. Ceramic structures were obtained after heat treatment in argon at 1300°C.

### Mechanical testing

Compression tests of EDCs in the form of 3D-printed microlattices or honeycombs were performed using an MTS Alliance RT/30 or MTS 810 testing machine, and the displacement rate was 0.5 mm min^−1^. Samples were bonded to steel sheets with glues before polishing and compression. An extensometer was used to measure the displacement of the sample in the compression. The compressive strengths of the structures were defined as the maximum load before collapse over the nominal cross-sectional area of the polished top surface. Tables S1 and S2 list the detailed information of all the samples in compression tests.

Tension tests of precursors in the form of 3D-printed solid cuboid (75 mm × 10 mm × 1 mm) were performed using the Tinius Olsen #H50KT Material Testing System, and the displacement rate was 50 mm min^−1^. Movies S1 and S2 show the tension testing of precursor printing by ink system 1 and ink system 2.

### Characterization

SEM (Quanta 450 FEG, FEI) was used to observe the unit cell of microlattices. The samples were coated with gold using a sputtering system (Q150 T S, Quorum Technologies Ltd.) before SEM characterization. To characterize porous structures inside EDCs, NOVA 1200e (Quantachrome Instruments) was used to obtain N_2_ absorption-desorption isotherms of EDCs. BET analysis was used to calculate the specific surface area of EDCs. To obtain the pore size distribution of EDCs, nonlinear density functional theory was used, and fitting errors were below 0.5%. To characterize the size and the atomic structure of ZrO_2_ NPs, TEM images were obtained. To characterize structures of EDCs, the TEM foils with a thickness of ~20 nm were prepared by using focused ion beam. A JEM 2100F FEG TEM (JEOL) operated at 200 kV was used for TEM analysis. The average composition of resultant EDCs was measured by EDS.

### Simulation

A 3D FEA was performed to simulate the evolution of the Miura-ori structure driven by compressive buckling. The commercial software Abaqus was used to conduct the simulations. The ceramic precursors, substrate, and joints were modeled as solid bodies because the lateral dimensions of the ceramic precursors and substrate were much larger than the lattice space in experiments. The finite element model is shown in fig. S5. Approximately 12,000 second-order hybrid elements (C3D20RH) were used during the simulations, and the convergence was examined. The dimension and size of the finite element model were the same as those in our experiments. The ceramic precursors and substrate were tied up with each other through the intermediate joints. In the analysis, initial geometric imperfections were introduced to the mesh to better trigger the deformation behavior of the structure. Moduli and Poisson’s ratios of ceramic precursors, substrate, and joints were all taken as 1 MPa and 0.5, respectively.

During the simulation of self-shaping assembly of the bending configuration, helical ribbon, and saddle surface, approximately 10,000 second-order hybrid elements (C3D20RH) were used. The finite element model was shown in Fig. 6, the dimensions of which were the same as those in experiments as well. Moduli of the ceramic precursors and printed patterns were measured by the Tinius Olsen #H50KT Material Testing System, and the values were 0.65 and 1.66 MPa, respectively. The Poisson’s ratios were both taken as 0.5.

## Supplementary Material

http://advances.sciencemag.org/cgi/content/full/4/8/eaat0641/DC1

## SUPPLEMENTARY MATERIALS

Supplementary material for this article is available at http://advances.sciencemag.org/cgi/content/full/4/8/eaat0641/DC1 Fig. S1. TEM image of ZrO_2_ NPs. Fig. S2. The effect of ZrO_2_ NPs on the ceramization of precursors printed by two ink systems. Fig. S3. The samples of elastomer, first EDCs, second EDCs, and oxidation results of the elastomer for two ink systems. Fig. S4. Porosity of EDCs. Fig. S5. Schematic of Miura-ori with the definition of important geometric parameters (*l*_1_ = *l*_2_ = 9 mm, *c* = 1.8 mm, α = 75°) and relative locations of the patterned joints on the substrate. Fig. S6. Phase diagram (¼) of 4D printing of the Miura-ori with FEA simulation and elastomeric experimental results. Fig. S7. Comparison of ink system 1 and ink system 2 with some different advantages. Table S1. Compression test samples with various conditions. Table S2. Compression test samples in Fig. 4 (ink system 1, first EDCs from heat treatment in argon at 1300°C). Movie S1. Tension testing video of precursors (played at 10× speed) printed by ink system 1. Movie S2. Tension testing video of precursors (played at 10× speed) printed by ink system 2. Movie S3. 4D printing of ceramic Miura-ori. Movie S4. FEA simulation showing shape morphing of the bending configuration. Movie S5. FEA simulation showing shape morphing of the helical ribbon. Movie S6. FEA simulation showing shape morphing of the saddle surface.

## References

[1] Py C., Reverdy P., Doppler L., Bico J., Roman B., Baroud C. N., Capillary origami: Spontaneous wrapping of a droplet with an elastic sheet. Phys. Rev. Lett. 98, 156103 (2007). 17501365 10.1103/PhysRevLett.98.156103

[2] Xu S., Yan Z., Jang K.-I., Huang W., Fu H., Kim J., Wei Z., Flavin M., McCracken J., Wang R., Badea A., Liu Y., Xiao D., Zhou G., Lee J., Chung H. U., Cheng H., Ren W., Banks A., Li X., Paik U., Nuzzo R. G., Huang Y., Zhang Y., Rogers J. A., Assembly of micro/nanomaterials into complex, three-dimensional architectures by compressive buckling. Science 347, 154–159 (2015). 25574018 10.1126/science.1260960

[3] Zhao Q., Zou W., Luo Y., Xie T., Shape memory polymer network with thermally distinct elasticity and plasticity. Sci. Adv. 2, e1501297 (2016). 26824077 10.1126/sciadv.1501297 PMC4730863

[4] Zhao Z., Wu J., Mu X., Chen H., Jerry Qi H., Fang D., Origami by frontal photopolymerization. Sci. Adv. 3, e1602326 (2017). 28508038 10.1126/sciadv.1602326 PMC5409495

[5] Felton S., Tolley M., Demaine E., Rus D., Wood R., A method for building self-folding machines. Science 345, 644–646 (2014). 25104380 10.1126/science.1252610

[6] Andersen E. S., Dong M., Nielsen M. M., Jahn K., Subramani R., Mamdouh W., Golas M. M., Sander B., Stark H., Oliveira C. L. P., Pedersen J. S., Birkedal V., Besenbacher F., Gothelf K. V., Kjems J., Self-assembly of a nanoscale DNA box with a controllable lid. Nature 459, 73–76 (2009). 19424153 10.1038/nature07971

[7] Jakus A. E., Rutz A. L., Jordan S. W., Kannan A., Mitchell S. M., Yun C., Koube K. D., Yoo S. C., Whiteley H. E., Richter C.-P., Galiano R. D., Hsu W. K., Stock S. R., Hsu E. L., Shah R. N., Hyperelastic “bone”: A highly versatile, growth factor–free, osteoregenerative, scalable, and surgically friendly biomaterial. Sci. Transl. Med. 8, 358ra127 (2016). 10.1126/scitranslmed.aaf7704 27683552

[8] Gladman A. S., Matsumoto E. A., Nuzzo R. G., Mahadevan L., Lewis J. A., Biomimetic 4D printing. Nat. Mater. 15, 413–418 (2016). 26808461 10.1038/nmat4544

[9] Ding Z., Yuan C., Peng X., Wang T., Qi H. J., Dunn M. L., Direct 4D printing via active composite materials. Sci. Adv. 3, e1602890 (2017). 28439560 10.1126/sciadv.1602890 PMC5389747

[10] Ahn B. Y., Shoji D., Hansen C. J., Hong E., Dunand D. C., Lewis J. A., Printed origami structures. Adv. Mater. 22, 2251–2254 (2010). 20397151 10.1002/adma.200904232

[11] Bargardi F. L., Le Ferrand H., Libanori R., Studart A. R., Bio-inspired self-shaping ceramics. Nat. Commun. 7, 13912 (2016). 28008930 10.1038/ncomms13912 PMC5196359

[12] Annett J., Cross G. L. W., Self-assembly of graphene ribbons by spontaneous self-tearing and peeling from a substrate. Nature 535, 271–275 (2016). 27411633 10.1038/nature18304

[13] Ionescu E., Kleebe H.-J., Riedel R., Silicon-containing polymer-derived ceramic nanocomposites (PDC-NCs): Preparative approaches and properties. Chem. Soc. Rev. 41, 5032–5052 (2012). 22415516 10.1039/c2cs15319j

[14] Eckel Z. C., Zhou C., Martin J. H., Jacobsen A. J., Carter W. B., Schaedler T. A., Additive manufacturing of polymer-derived ceramics. Science 351, 58–62 (2016). 26721993 10.1126/science.aad2688

[15] Zanchetta E., Cattaldo M., Franchin G., Schwentenwein M., Homa J., Brusatin G., Colombo P., Stereolithography of SiOC ceramic microcomponents. Adv. Mater. 28, 370–376 (2016). 26545292 10.1002/adma.201503470

[16] Kotz F., Arnold K., Bauer W., Schild D., Keller N., Sachsenheimer K., Nargang T. M., Richter C., Helmer D., Rapp B. E., Three-dimensional printing of transparent fused silica glass. Nature 544, 337–339 (2017). 28425999 10.1038/nature22061

[17] Truby R. L., Lewis J. A., Printing soft matter in three dimensions. Nature 540, 371–378 (2016). 27974748 10.1038/nature21003

[18] Hammock M. L., Chortos A., Tee B. C.-K., Tok J. B.-H., Bao Z., 25th anniversary article: The evolution of electronic skin (e-skin): A brief history, design considerations, and recent progress. Adv. Mater. 25, 5997–6038 (2013). 24151185 10.1002/adma.201302240

[19] Hamdani S., Longuet C., Perrin D., Lopez-cuesta J.-M., Ganachaud F., Flame retardancy of silicone-based materials. Polym. Degrad. Stab. 94, 465–495 (2009).

[20] Wehner M., Truby R. L., Fitzgerald D. J., Mosadegh B., Whitesides G. M., Lewis J. A., Wood R. J., An integrated design and fabrication strategy for entirely soft, autonomous robots. Nature 536, 451–455 (2016). 27558065 10.1038/nature19100

[21] Rogers J. A., Someya T., Huang Y., Materials and mechanics for stretchable electronics. Science 327, 1603–1607 (2010). 20339064 10.1126/science.1182383

[22] Kashiwagi T., Du F., Douglas J. F., Winey K. I., Harris R. H. Jr., Shields J. R., Nanoparticle networks reduce the flammability of polymer nanocomposites. Nat. Mater. 4, 928–933 (2005). 16267575 10.1038/nmat1502

[23] Colombo P., Mera G., Riedel R., Sorarù G. D., Polymer-derived ceramics: 40 years of research and innovation in advanced ceramics. J. Am. Ceram. Soc. 93, 1805–1837 (2010).

[24] Klein Y., Efrati E., Sharon E., Shaping of elastic sheets by prescription of non-Euclidean metrics. Science 315, 1116–1120 (2007). 17322058 10.1126/science.1135994

[25] Aharoni H., Sharon E., Kupferman R., Geometry of thin nematic elastomer sheets. Phys. Rev. Lett. 113, 257801 (2014). 25554907 10.1103/PhysRevLett.113.257801

[26] Hathavway A. S., General investigations of curved surfaces. Science 16, 902–903 (1902).

[27] Dudte L. H., Vouga E., Tachi T., Mahadevan L., Programming curvature using origami tessellations. Nat. Mater. 15, 583–588 (2016). 26808459 10.1038/nmat4540

[28] MacDonald E., Wicker R., Multiprocess 3D printing for increasing component functionality. Science 353, aaf2093 (2016). 27708075 10.1126/science.aaf2093

[29] Blees M. K., Barnard A. W., Rose P. A., Roberts S. P., McGill K. L., Huang P. Y., Ruyack A. R., Kevek J. W., Kobrin B., Muller D. A., McEuen P. L., Graphene kirigami. Nature 524, 204–207 (2015). 26222025 10.1038/nature14588

[30] Silverberg J. L., Evans A. A., McLeod L., Hayward R. C., Hull T., Santangelo C. D., Cohen I., Using origami design principles to fold reprogrammable mechanical metamaterials. Science 345, 647–650 (2014). 25104381 10.1126/science.1252876

[31] Armon S., Efrati E., Kupferman R., Sharon E., Geometry and mechanics in the opening of chiral seed pods. Science 333, 1726–1730 (2011). 21940888 10.1126/science.1203874

[32] Pikul J. H., Li S., Bai H., Hanlon R. T., Cohen I., Shepherd R. F., Stretchable surfaces with programmable 3D texture morphing for synthetic camouflaging skins. Science 358, 210–214 (2017). 29026040 10.1126/science.aan5627

[33] Meza L. R., Das S., Greer J. R., Strong, lightweight, and recoverable three-dimensional ceramic nanolattices. Science 345, 1322–1326 (2014). 25214624 10.1126/science.1255908

[34] Bauer J., Hengsbach S., Tesari I., Schwaiger R., Kraft O., High-strength cellular ceramic composites with 3D microarchitecture. Proc. Natl. Acad. Sci. U.S.A. 111, 2453–2458 (2014). 24550268 10.1073/pnas.1315147111 PMC3932926

[35] Bauer J., Schroer A., Schwaiger R., Kraft O., Approaching theoretical strength in glassy carbon nanolattices. Nat. Mater. 15, 438–443 (2016). 26828314 10.1038/nmat4561

[36] Colombo P., Hellmann J. R., Shelleman D. L., Mechanical properties of silicon oxycarbide ceramic foams. J. Am. Ceram. Soc. 84, 2245–2251 (2001).

[37] Zheng X., Smith W., Jackson J., Moran B., Cui H., Chen D., Ye J., Fang N., Rodriguez N., Weisgraber T., Spadaccini C. M., Multiscale metallic metamaterials. Nat. Mater. 15, 1100–1106 (2016). 27429209 10.1038/nmat4694

[38] Padture N. P., Advanced structural ceramics in aerospace propulsion. Nat. Mater. 15, 804–809 (2016). 27443899 10.1038/nmat4687

[39] Munch E., Launey M. E., Alsem D. H., Saiz E., Tomsia A. P., Ritchie R. O., Tough, bio-inspired hybrid materials. Science 322, 1516–1520 (2008). 19056979 10.1126/science.1164865

[40] Mao L.-B., Gao H.-L., Yao H.-B., Liu L., Cölfen H., Liu G., Chen S.-M., Li S.-K., Yan Y.-X., Liu Y.-Y., Yu S.-H., Synthetic nacre by predesigned matrix-directed mineralization. Science 354, 107–110 (2016). 27540008 10.1126/science.aaf8991

