# Supplementary material for: Origami and 4D printing of elastomer-derived ceramic structures
Source: Sci Adv. 2018 Aug 17;4(8):eaat0641. doi: 10.1126/sciadv.aat0641 (PMC6097816; doi:10.1126/sciadv.aat0641)
Supplement: http://advances.sciencemag.org/cgi/content/full/4/8/eaat0641/DC1 [file supp_4_8_eaat0641__index.html]

Science Advances | Science Advances

## Supplementary Materials

**The PDF file includes:**

- Fig. S1. TEM image of ZrO2 NPs.
- Fig. S2. The effect of ZrO2 NPs on the ceramization of precursors printed by two ink systems.
- Fig. S3. The samples of elastomer, first EDCs, second EDCs, and oxidation results of the elastomer for two ink systems.
- Fig. S4. Porosity of EDCs.
- Fig. S5. Schematic of Miura-ori with the definition of important geometric parameters (*l*1 = *l*2 = 9 mm, *c* = 1.8 mm, α = 75°) and relative locations of the patterned joints on the substrate.
- Fig. S6. Phase diagram (¼) of 4D printing of the Miura-ori with FEA simulation and elastomeric experimental results.
- Fig. S7. Comparison of ink system 1 and ink system 2 with some different advantages.
- Table S1. Compression test samples with various conditions.
- Table S2. Compression test samples in Fig. 4 (ink system 1, first EDCs from heat treatment in argon at 1300°C).
- Legends for movies S1 to S6.

Download PDF

**Other Supplementary Material for this manuscript includes the following:**

- Movie S1 (.mp4 format). Tension testing video of precursors (played at 10× speed) printed by ink system 1.
- Movie S2 (.mp4 format). Tension testing video of precursors (played at 10× speed) printed by ink system 2.
- Movie S3 (.mp4 format). 4D printing of ceramic Miura-ori.
- Movie S4 (.mp4 format). FEA simulation showing shape morphing of the bending configuration.
- Movie S5 (.mp4 format). FEA simulation showing shape morphing of the helical ribbon.
- Movie S6 (.mp4 format). FEA simulation showing shape morphing of the saddle surface.

**Files in this Data Supplement:**

- Adobe PDF - aat0641\_SM.pdf
