# Supplementary material for: Origami and 4D printing of elastomer-derived ceramic structures
Source: Sci Adv. 2018 Aug 17;4(8):eaat0641. doi: 10.1126/sciadv.aat0641 (PMC6097816; doi:10.1126/sciadv.aat0641)
Supplement: http://advances.sciencemag.org/cgi/content/full/4/8/eaat0641/DC1 [file aat0641_SM.pdf]

Science Advances
AAAS

advances.sciencemag.org/cgi/content/full/4/8/eaat0641/DC1

Supplementary Materials for

Origami and 4D printing of elastomer-derived ceramic structures

Guo Liu, Yan Zhao, Ge Wu, Jian Lu*

*Corresponding author. Email: jianlu@cityu.edu.hk

Published 17 August 2018, *Sci. Adv.* **4**, eaat0641 (2018)
DOI: 10.1126/sciadv.aat0641

The PDF file includes:

Fig. S1. TEM image of ZrO_2 NPs.
Fig. S2. The effect of ZrO_2 NPs on the ceramization of precursors printed by two ink systems.
Fig. S3. The samples of elastomer, first EDCs, second EDCs, and oxidation results of the elastomer for two ink systems.
Fig. S4. Porosity of EDCs.
Fig. S5. Schematic of Miura-ori with the definition of important geometric parameters ($l_1 = l_2 = 9$ mm, $c = 1.8$ mm, $\alpha = 75°$) and relative locations of the patterned joints on the substrate.
Fig. S6. Phase diagram ($^1/_4$) of 4D printing of the Miura-ori with FEA simulation and elastomeric experimental results.
Fig. S7. Comparison of ink system 1 and ink system 2 with some different advantages.
Table S1. Compression test samples with various conditions.
Table S2. Compression test samples in Fig. 4 (ink system 1, first EDCs from heat treatment in argon at 1300°C).
Legends for movies S1 to S6.

Other Supplementary Material for this manuscript includes the following:

(available at advances.sciencemag.org/cgi/content/full/4/8/eaat0641/DC1)

Movie S1 (.mp4 format). Tension testing video of precursors (played at 10× speed) printed by ink system 1.
Movie S2 (.mp4 format). Tension testing video of precursors (played at 10× speed) printed by ink system 2.
Movie S3 (.mp4 format). 4D printing of ceramic Miura-ori.
Movie S4 (.mp4 format). FEA simulation showing shape morphing of the bending configuration.
Movie S5 (.mp4 format). FEA simulation showing shape morphing of the helical ribbon.
Movie S6 (.mp4 format). FEA simulation showing shape morphing of the saddle surface.

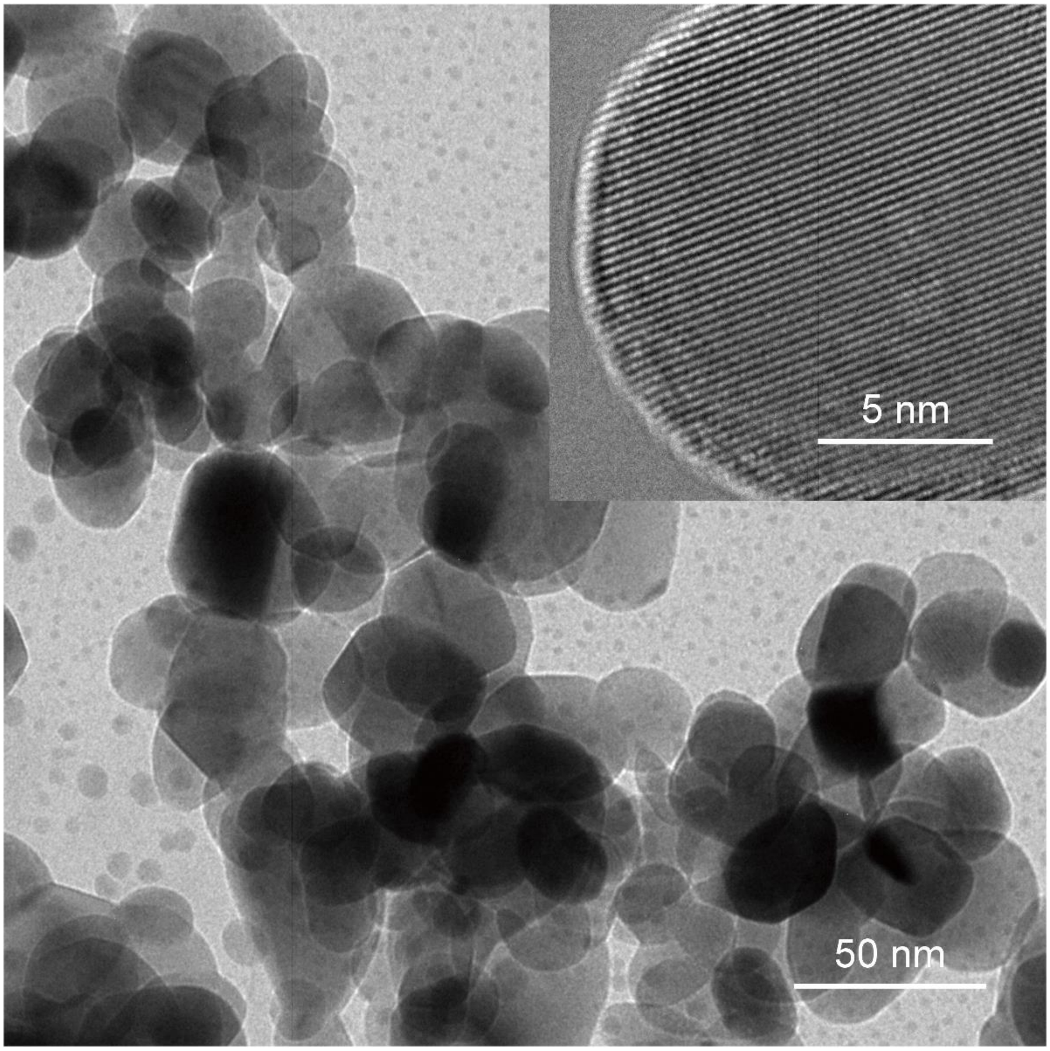

Fig. S1. TEM image of ZrO_2 NPs. The inset shows high-resolution imaging results.

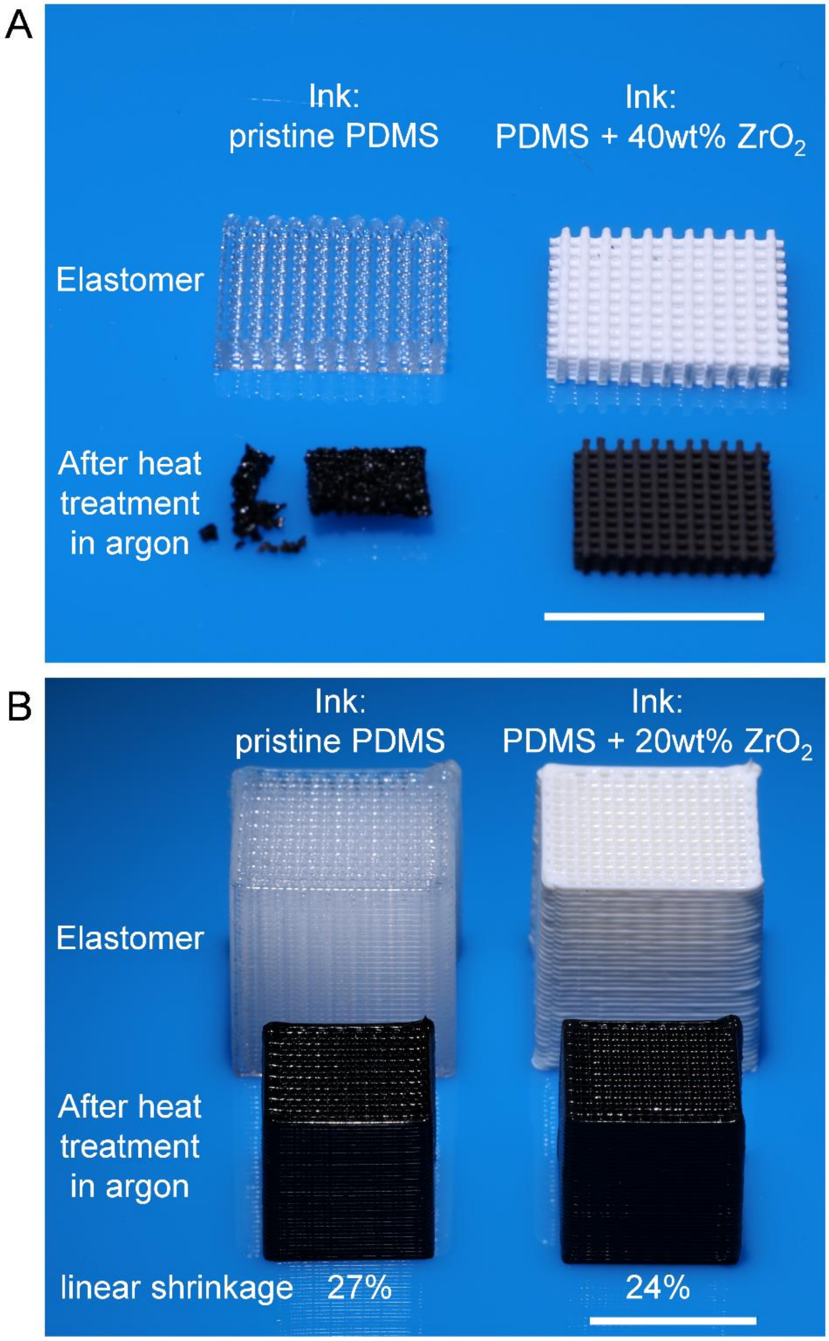

Fig. S2. The effect of ZrO_2 NPs on the ceramization of precursors printed by two ink systems. For ink system 1 (**A**), heating of pristine PDMS in argon showed poor structural stability. For ink system 2 (**B**), ZrO_2-filled PDMS showed eliminated shrinkage upon ceramization. Scale bars, 1cm.

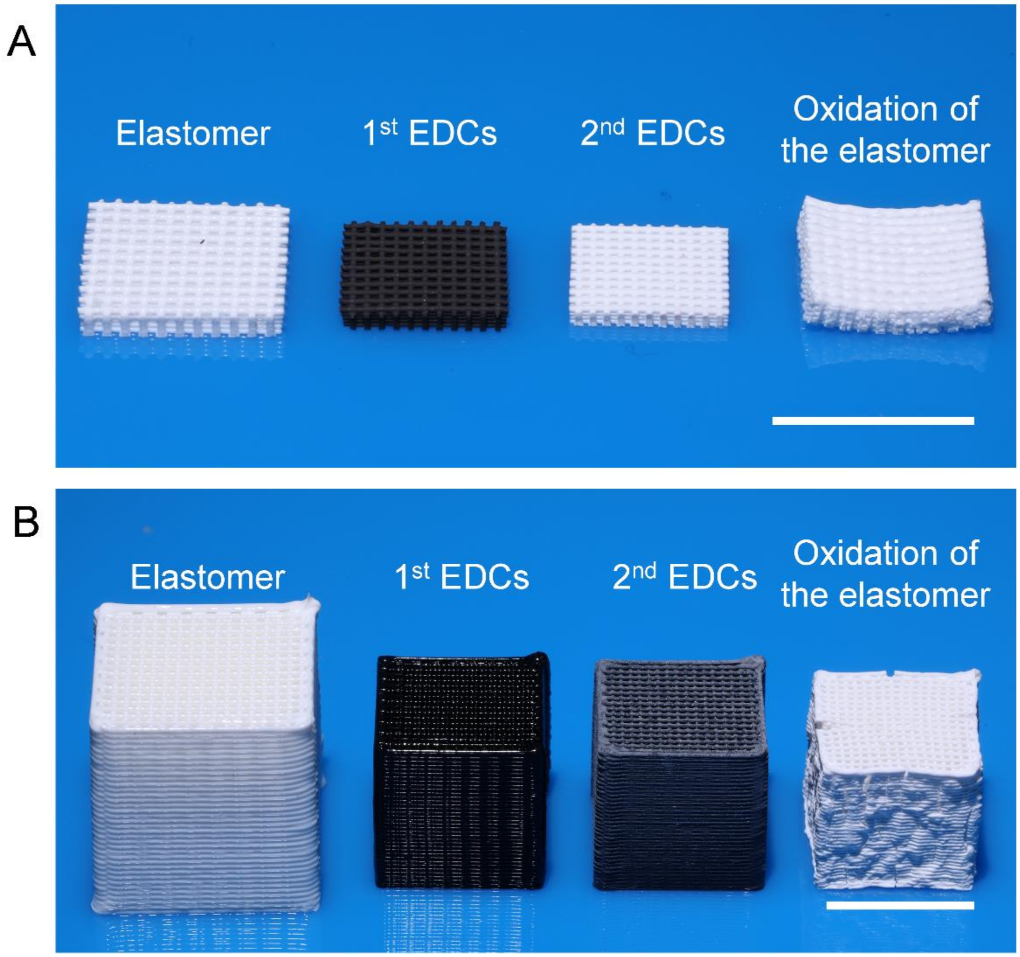

Fig. S3. The samples of elastomer, first EDCs, second EDCs, and oxidation results of the elastomer for two ink systems. For ink system 1 (**A**) and ink system 2 (**B**), the oxidation results of the elastomer demonstrated poor structural retention and mechanical integrity. Scale bars, 1cm.

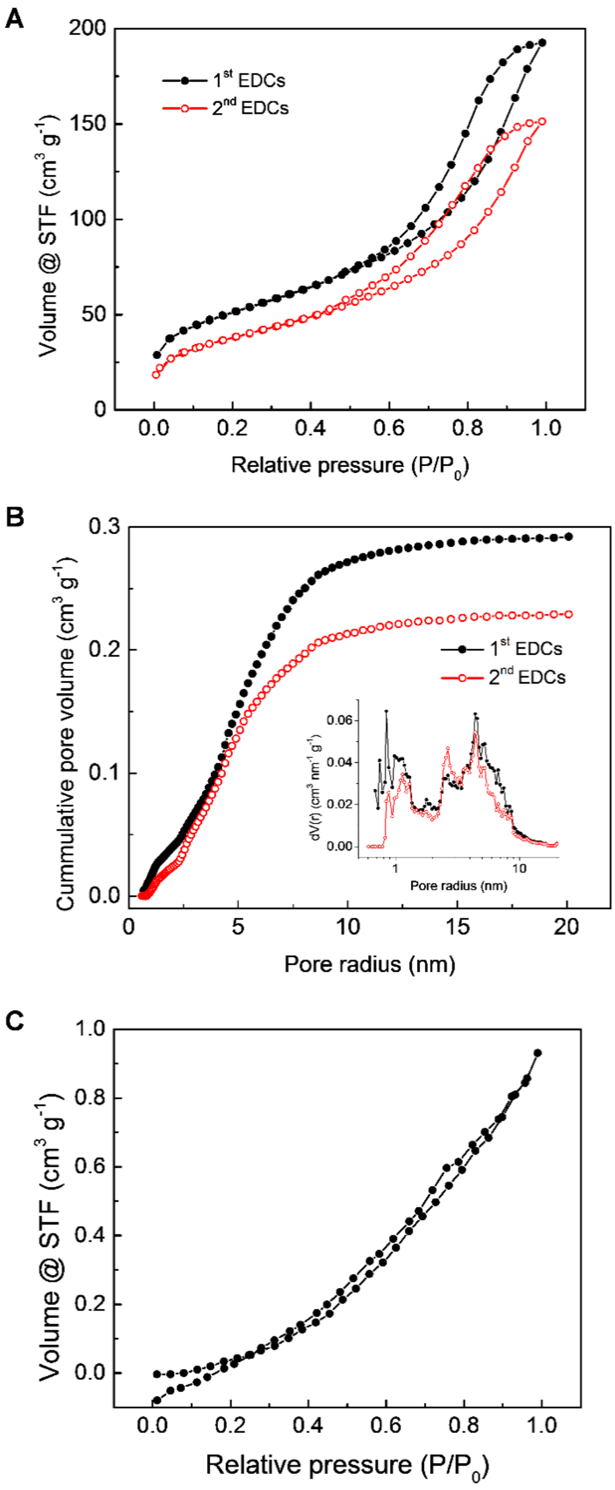

Fig. S4. Porosity of EDCs. (**A**) N_2 adsorption/desorption isotherm of first and second EDCs. These first EDCs were obtained by heat treatment of Ink System 1 in 1000°C under argon flow. (**B**) Cumulative pore volume and pore size distribution (inset) of first and second EDCs. These first EDCs were obtained by heat treatment of Ink System 1 in 1000°C under argon flow. (**C**) N_2 adsorption/desorption isotherm of first EDCs obtained by heat treatment of Ink System 2 in 1300°C under argon flow.

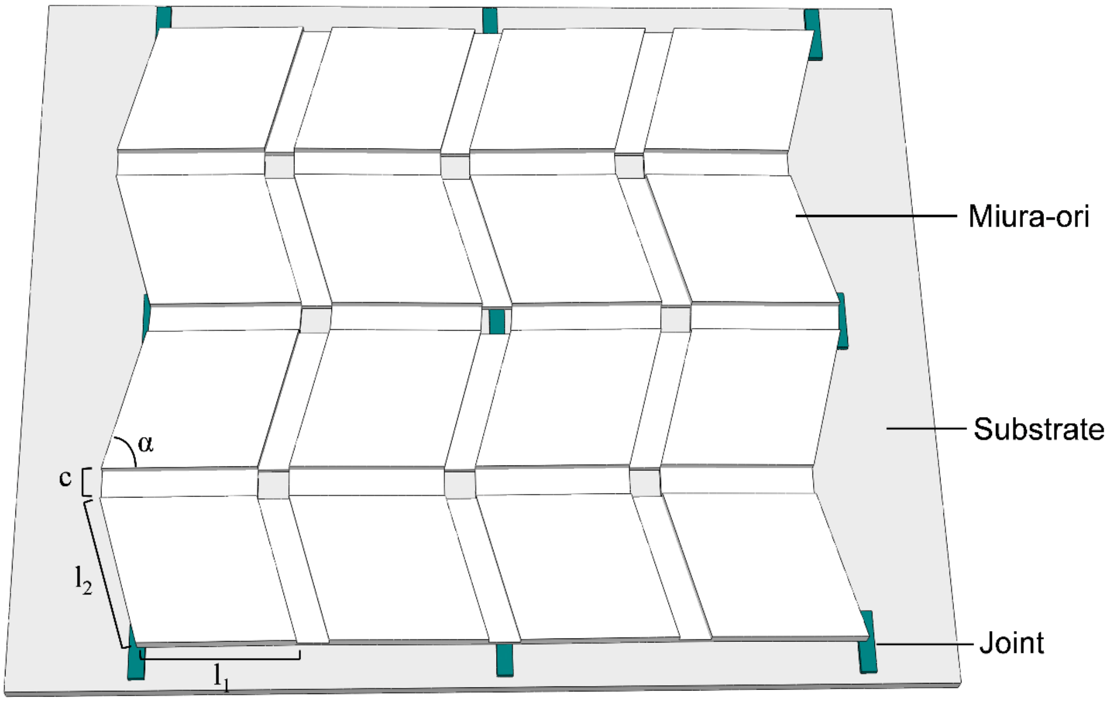

Fig. S5. Schematic of Miura-ori with the definition of important geometric parameters (l_1 = l_2 = 9 mm, *c* = 1.8 mm, α = 75°) and relative locations of the patterned joints on the substrate.

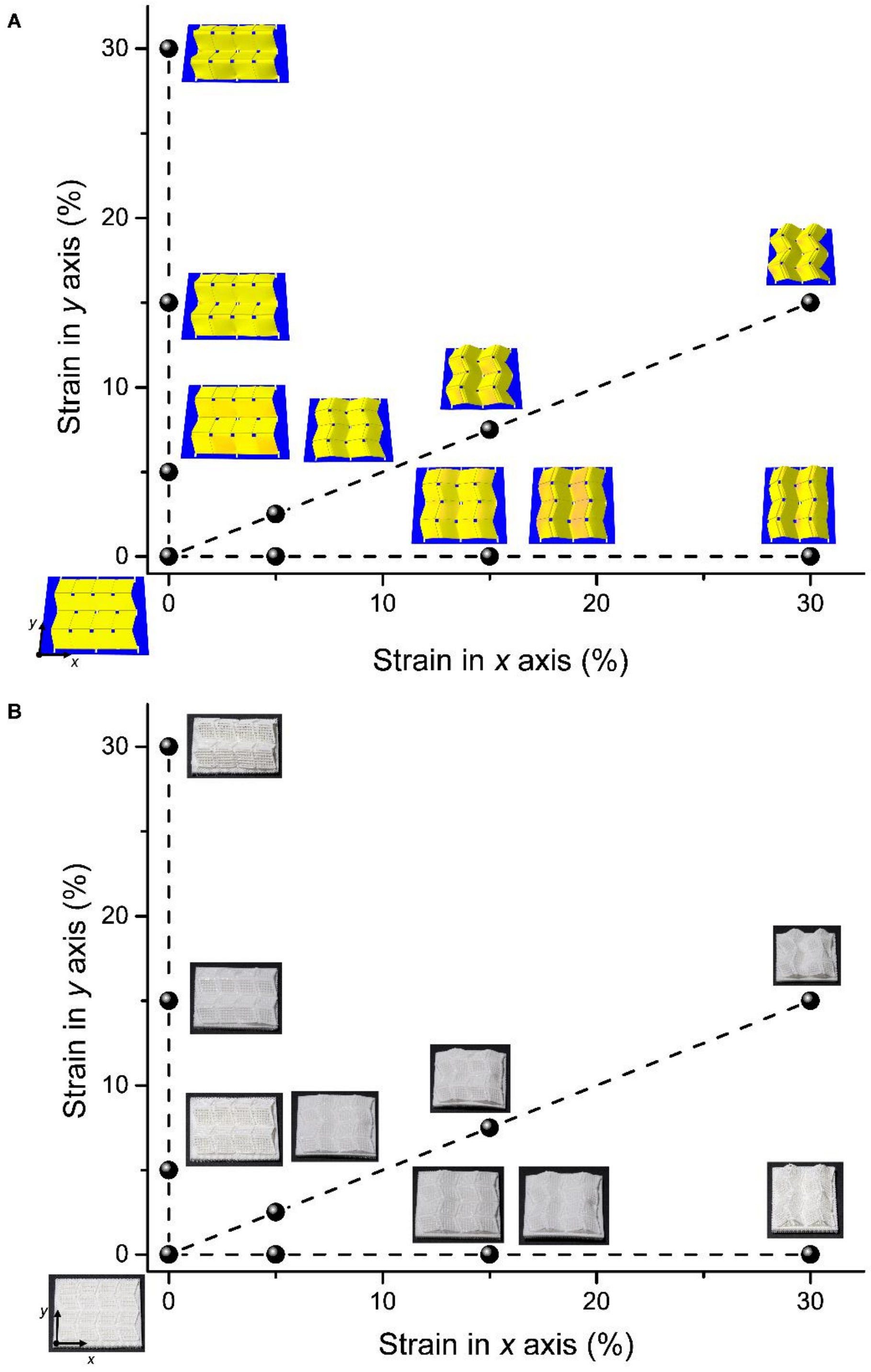

Fig. S6. Phase diagram ($^{1}/_{4}$) of 4D printing of the Miura-ori with FEA simulation and elastomeric experimental results. The FEA simulation (A) and elastomeric experimental results (B) of Miura-ori is an example to illustrate that a series of complex-shaped ceramics with continuously variable geometries can be derived from a simple design.

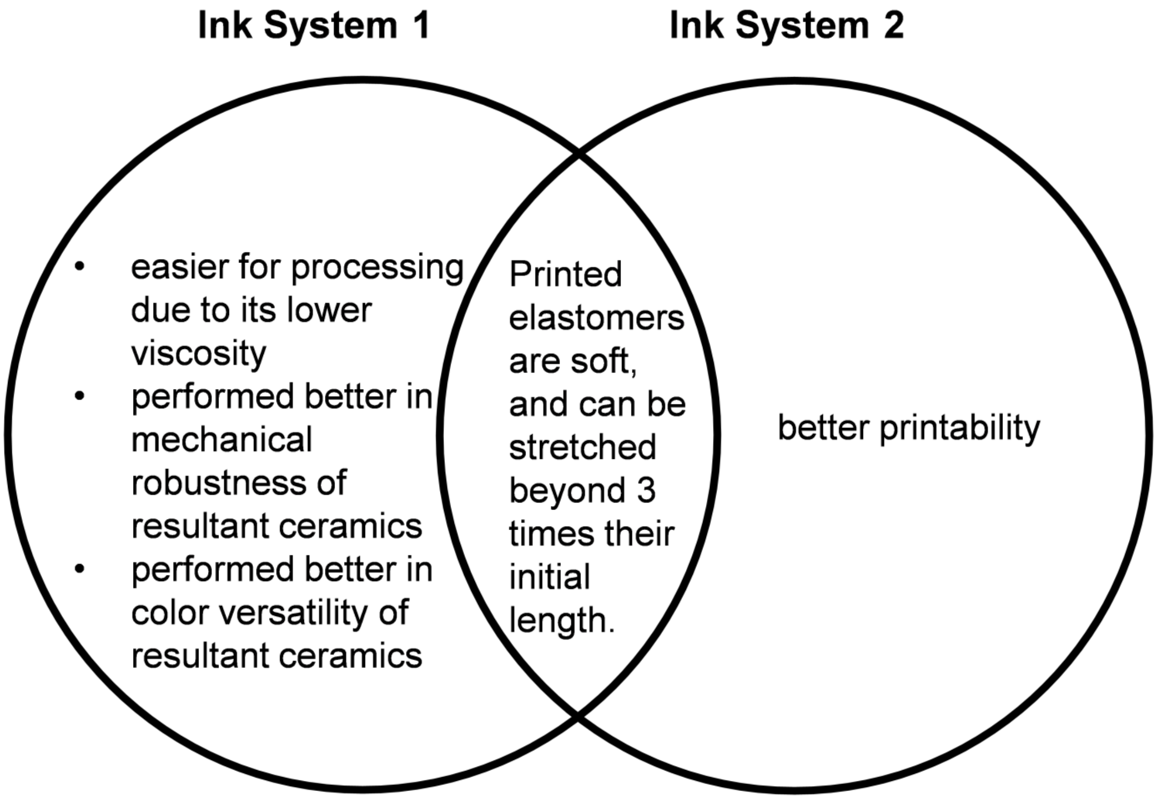

Fig. S7. Comparison of ink system 1 and ink system 2 with some different advantages.

Table S1. Compression test samples with various conditions.

Ink	Material	Maximum temperature in heat treatment for 1st EDCs (°C)	Atmosphere in heat treatment for 1st EDCs	Architecture	Geometry	d (μm)	a (mm)	b (mm)	Dimensions of the precursor* (mm)	Mass of the precursor (g)	Dimensions of the sample (mm)	Mass of the sample (g)	Density (g cm^{-3})	Compressive strength (MPa)
System 1	1st EDCs	1000	Vacuum	Lattice	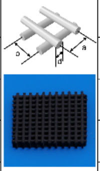	208	0.64	0.64	10.40x10.38 x1.59	0.162	8.40x8.40 x1.30	0.108	1.18	205.8
System 1	1st EDCs	1000	Vacuum	Lattice		208	0.64	0.64	10.32x10.32 x1.52	0.151	8.33x8.33 x1.25	0.099	1.14	160.4
System 1	1st EDCs	1000	Vacuum	Lattice		208	0.64	0.64	10.50x10.50 x1.54	0.136	8.44x8.42 x1.21	0.089	1.04	150.2
System 1	1st EDCs	1000	Argon	Lattice		208	0.64	0.64	10.72x10.61 x1.60	0.173	8.62x8.52 x1.31	0.112	1.16	207.0
System 1	1st EDCs	1000	Argon	Lattice		208	0.64	0.64	10.72x10.59 x1.51	0.155	8.57x8.49 x1.21	0.100	1.14	211.2
System 1	1st EDCs	1000	Argon	Lattice		208	0.64	0.64	10.70x10.58 x1.46	0.148	8.62x8.42 x1.20	0.094	1.08	174.0
System 1	1st EDCs	1000	Argon	Honeycomb	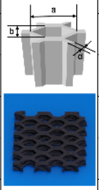	208	2.25	1.30	11.02x10.25 x1.93	0.086	8.96x8.30 x1.55	0.057	0.49	34.2
System 1	1st EDCs	1000	Argon	Honeycomb		208	2.25	1.30	11.06x10.21 x1.66	0.088	8.85x8.28 x1.36	0.057	0.57	53.9
System 1	1st EDCs	1000	Argon	Honeycomb		208	2.25	1.30	11.20x10.49 x1.58	0.080	8.98x8.54 x1.25	0.052	0.54	58.6
System 1	1st EDCs	1000	Argon	Honeycomb		208	2.25	1.30	10.95x10.29 x2.30	0.135	8.90x8.39 x1.83	0.087	0.64	41.6
System 1	1st EDCs	1000	Argon	Honeycomb		208	2.25	1.30	11.13x10.02 x2.12	0.116	8.92x8.10 x1.68	0.075	0.62	46.8
System 1	1st EDCs	1000	Argon	Honeycomb		208	2.25	1.30	10.86x10.07 x2.38	0.145	8.74x8.18 x1.93	0.095	0.69	42.9
System 1	2nd EDCs	1000	Vacuum	Lattice	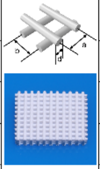	203	0.63	0.63	8.25x8.25 x1.26	0.095	8.01x8.01 x1.21	0.092	1.19	172.2
System 1	2nd EDCs	1000	Vacuum	Lattice		203	0.63	0.63	8.40x8.34 x1.23	0.093	8.11x8.24 x1.18	0.090	1.14	150.7
System 1	2nd EDCs	1000	Vacuum	Lattice		203	0.63	0.63	8.38x8.42 x1.27	0.097	8.20x8.16 x1.22	0.095	1.16	175.2
System 1	2nd EDCs	1000	Argon	Lattice		203	0.63	0.63	8.64x8.51 x1.26	0.099	8.43x8.31 x1.23	0.098	1.14	169.3
System 1	2nd EDCs	1000	Argon	Lattice		203	0.63	0.63	8.59x8.51 x1.24	0.101	8.40x8.30 x1.22	0.100	1.18	182.9
System 1	2nd EDCs	1000	Argon	Lattice		203	0.63	0.63	8.57x8.53 x1.16	0.091	8.40x8.34 x1.15	0.090	1.12	169.2
System 1	2nd EDCs	1000	Argon	Honeycomb	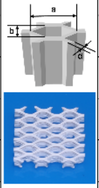	203	2.20	1.27	8.89x8.24 x1.29	0.056	8.71x8.15 x1.25	0.054	0.61	74.3
System 1	2nd EDCs	1000	Argon	Honeycomb		203	2.20	1.27	9.05x8.53 x1.35	0.053	8.90x8.31 x1.33	0.051	0.52	57.7
System 1	2nd EDCs	1000	Argon	Honeycomb		203	2.20	1.27	9.02x8.52 x1.34	0.060	8.80x8.30 x1.33	0.057	0.59	57.3
System 1	2nd EDCs	1000	Argon	Honeycomb		203	2.20	1.27	8.84x8.14 x1.71	0.078	8.61x7.92 x1.66	0.076	0.67	55.9
System 1	2nd EDCs	1000	Argon	Honeycomb		203	2.20	1.27	8.84x8.36 x1.76	0.095	8.56x8.12 x1.71	0.094	0.79	75.1
System 1	2nd EDCs	1000	Argon	Honeycomb		203	2.20	1.27	8.88x8.31 x1.79	0.093	8.60x8.05 x1.74	0.092	0.76	70.7
System 2	1st EDCs	1300	Argon	Lattice	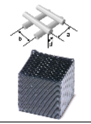	198	0.61	0.61	14.46x14.41 x14.54	2.132	11.04x11.01 x11.09	1.697	1.26	221.5
System 2	1st EDCs	1300	Argon	Lattice		198	0.61	0.61	14.59x14.57 x14.62	2.422	11.17x11.21 x11.09	1.939	1.40	232.6
System 2	1st EDCs	1300	Argon	Lattice		198	0.61	0.61	14.56x14.53 x14.59	2.334	11.08x11.07 x11.03	1.854	1.37	267.1

* Precursor: PDMS NCs for 1st EDCs; 1st EDCs for 2nd EDCs

Table S2. Compression test samples in Fig. 4 (ink system 1, first EDCs from heat treatment in argon at 1300°C).

Geometry	d (μm)	a (mm)	b (mm)	Dimensions of the sample (mm)	Mass of the sample (g)	Density (g cm^{-3})	Compressive strength (MPa)	Specific compressive strength (MPa cm^3 g^{-1})
	203	0.62	0.62	8.18x8.13x1.22	0.124	1.53	333.6	218.0
	203	0.62	0.62	8.15x8.10x1.34	0.121	1.36	301.6	221.0
	203	0.62	0.62	8.13x8.09x1.31	0.114	1.32	298.4	225.5
	203	0.62	0.62	8.13x8.00x1.32	0.119	1.38	316.4	229.8
	203	0.62	0.62	7.74x7.71x1.13	0.117	1.74	453.8	260.8
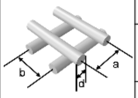	203	0.62	0.62	8.14x8.07x1.34	0.130	1.48	385.9	261.5
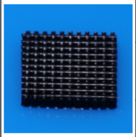	203	0.62	0.62	7.90x7.84x1.20	0.127	1.70	484.9	285.2
	203	0.62	0.62	7.97x7.78x1.21	0.122	1.63	474.3	291.0
	203	0.62	0.62	7.82x7.72x1.20	0.124	1.71	508.2	297.2
	203	0.62	0.62	8.06x7.99x1.16	0.111	1.49	449.4	301.6
	203	0.62	0.62	7.84x7.83x1.20	0.120	1.63	496.9	304.8
	203	0.62	0.62	8.07x7.99x1.21	0.113	1.45	455.8	314.3
	203	0.62	0.62	8.11x7.95x1.23	0.117	1.48	496.3	335.3
	203	0.62	0.62	7.917.90x1.14	0.116	1.62	546.7	337.4
	203	0.62	0.62	8.03x7.96x1.20	0.112	1.46	495.7	339.2

Movie S1. Tension testing video of precursors (played at 10× speed) printed by ink system 1. The precursors can be stretched to at least 200% strain.

Movie S2. Tension testing video of precursors (played at 10× speed) printed by ink system 2. The precursors can be stretched to at least 200% strain.

Movie S3. 4D printing of ceramic Miura-ori. The movie shows 4D printing process of the ceramic Miura-ori, including 3D printing of precursors (played at 10x speed), 3D printing of the substrate (played at 15x speed), stretching of the substrate (played at 1x speed), printing joints on the substrate (played at 5x speed), compressive buckling of precursors (this video is at 1x playback speed), and synthesis of first and second EDCs.

Movie S4. FEA simulation showing shape morphing of the bending configuration.

Movie S5. FEA simulation showing shape morphing of the helical ribbon.

Movie S6. FEA simulation showing shape morphing of the saddle surface.
